# New sheathless supra-aortic pulsatile mechanical support via subclavian access for combined protected percutaneous coronary intervention and transcatheter aortic valve implantation in hostile vascular disease: a case report

**DOI:** 10.1093/ehjcr/ytag502

**Published:** 2026-07-10

**Authors:** Edoardo Elia, Marco Lombardi, Giovanni La Malfa, Giuseppe Patti, Gioel Gabrio Secco

**Affiliations:** Division of Cardiology of IRCCS University Hospital SS Antonio e Biagio e Cesare Arrigo, Via Venezia 16, 15121 Alessandria, Italy; Division of Cardiology of IRCCS University Hospital SS Antonio e Biagio e Cesare Arrigo, Via Venezia 16, 15121 Alessandria, Italy; Division of Cardiology of IRCCS University Hospital SS Antonio e Biagio e Cesare Arrigo, Via Venezia 16, 15121 Alessandria, Italy; Department of Translational Medicine, University of Eastern Piedmont, Via Solaroli 17, 28100 Novara, Italy; Division of Cardiology, Maggiore della Carità Hospital, Corso Mazzini 18, 28100 Novara, Italy; Division of Cardiology of IRCCS University Hospital SS Antonio e Biagio e Cesare Arrigo, Via Venezia 16, 15121 Alessandria, Italy; Department of Translational Medicine, University of Eastern Piedmont, Via Solaroli 17, 28100 Novara, Italy

**Keywords:** Pulsatile mechanical circulatory support, Sheathless circulatory support, Complex structural and coronary intervention, Case report

## Abstract

**Background:**

The combination of severe aortic stenosis, complex multivessel coronary artery disease and severe left ventricular systolic dysfunction represents a procedural challenge in which coronary revascularization and transcatheter aortic valve implantation (TAVI) must be carefully integrated. Mechanical circulatory support (MCS) indication and selection are crucial when extensive peripheral arterial disease limits conventional large-bore access and when interaction with valve deployment must be avoided.

**Case summary:**

An 84-year-old man was admitted to our intensive cardiac care unit with acute decompensated heart failure. Echocardiography showed severely reduced left ventricular ejection fraction (15%) and severe aortic stenosis (mean gradient 41 mmHg; aortic valve area by velocity-time integral 0.45 cm^2^). Coronary angiography revealed critical stenoses of the left main (LM), proximal and mid left anterior descending artery (LAD), and ostial and proximal right coronary artery (RCA). Computed tomography revealed extensive aorto-iliac thrombosis, narrow sinotubular junction and short valve-to-coronary distances. Following Heart Team discussion, a combined strategy was adopted. After percutaneous transluminal angioplasty of right iliac artery from right femoral access, sheathless pulsatile MCS (iVAC-2L) was implanted percutaneously via left subclavian access and positioned above the aortic leaflets. Protected percutaneous coronary intervention (PCI) of LM–LAD was then performed. Transcatheter aortic valve implantation with a balloon-expandable valve followed and RCA PCI was subsequently completed. Mechanical circulatory support was maintained throughout all phases.

**Conclusion:**

The new sheathless pulsatile MCS provides haemodynamic stabilization, reduces access profile compared with the previous generation of the device and enables integration of complex PCI and transfemoral TAVI in patients with hostile vascular anatomy.

Learning points

- The dedicated sheathless iVAC-2L system enables mechanical circulatory support in patients with limited large-bore access.
- Supra-aortic support provides haemodynamic stability without interfering during transcatheter heart valve implantation.
- Minimizing vascular manipulation is key in extensive aorto-iliac thrombotic disease.

## Introduction

The coexistence of severe aortic stenosis, left main coronary artery (LM) disease, and advanced left ventricular dysfunction poses significant challenges in clinical decision-making and procedural planning in which revascularization and valve intervention are closely interdependent.^1^ Coronary treatment may precipitate instability, whereas valve replacement without prior revascularization may limit myocardial recovery. Mechanical circulatory support (MCS) can mitigate intra-procedural risk; however, many percutaneous systems require transvalvular left ventricular positioning, potentially interfering with transcatheter aortic valve implantation (TAVI).^2-4^ Concomitant severe aorto-iliac disease further complicates large-bore femoral access and increases vascular and embolic risk. The iVAC-2L provides pulsatile supra-aortic support without crossing the valve,^5^ and its newly developed sheathless 16F configuration reduces access requirements in hostile vascular anatomy.

To our knowledge, this represents the first reported clinical use of the novel dedicated sheathless iVAC-2L catheter within an integrated PCI–TAVI strategy in the setting of prohibitive aorto-iliac disease.

## Summary figure

**Figure:**
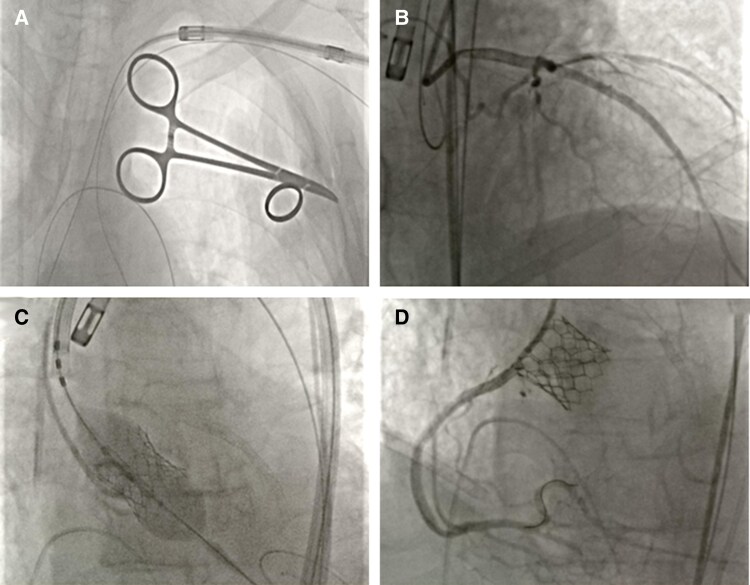
Trans-subclavian pulsatile support with the new dedicated sheathless iVAC-2L positioned above the aortic leaflets, allowing protected percutaneous coronary intervention and transcatheter aortic valve implantation. Panels are arranged from left to right and from top to bottom: *(A)* insertion of the dedicated sheathless iVAC-2L catheter; *(B)* result of left coronary artery percutaneous coronary intervention (provisional); *(C)* Sapien 3 Ultra 23 mm during rapid pacing; and *(D)* result of right coronary artery percutaneous coronary intervention.

## Case presentation

An 84-year-old man without known cardiovascular disease was admitted with acute pulmonary oedema and haemodynamic deterioration. On examination, he was in respiratory distress with bilateral crackles and a systolic ejection murmur. Laboratory tests showed elevated troponin and acute kidney injury on chronic kidney disease. Echocardiography revealed a severely dilated left ventricle with ejection fraction of 15% and severe calcific aortic stenosis (mean gradient 41 mmHg; valve area 0.45 cm^2^), with preserved right ventricular function and mild-to-moderate mitral regurgitation. Initial medical therapy with intravenous diuretics and dobutamine led to clinical improvement, with stable creatinine levels (3–4 mg/dL).

Coronary angiography demonstrated critical distal left main disease extending into the proximal and mid left anterior descending artery (LAD), and severe ostial and proximal right coronary artery (RCA) stenosis. Computed tomography showed extensive infrarenal aorto-iliac thrombosis, including right common iliac (RCI) occlusion and severe left iliac stenosis, along with a narrow sinotubular junction and short valve-to-coronary distances despite high coronary take-off (LM 19 mm, RCA 20 mm). Subclavian arteries were suitable for large-bore access (*Figure 1*). Surgical risk was prohibitive (STS mortality 22.1%; EuroSCORE II 59.56%). After Heart Team discussion, a fully percutaneous single-session PCI–TAVI strategy with MCS was selected.

**Figure 1:**
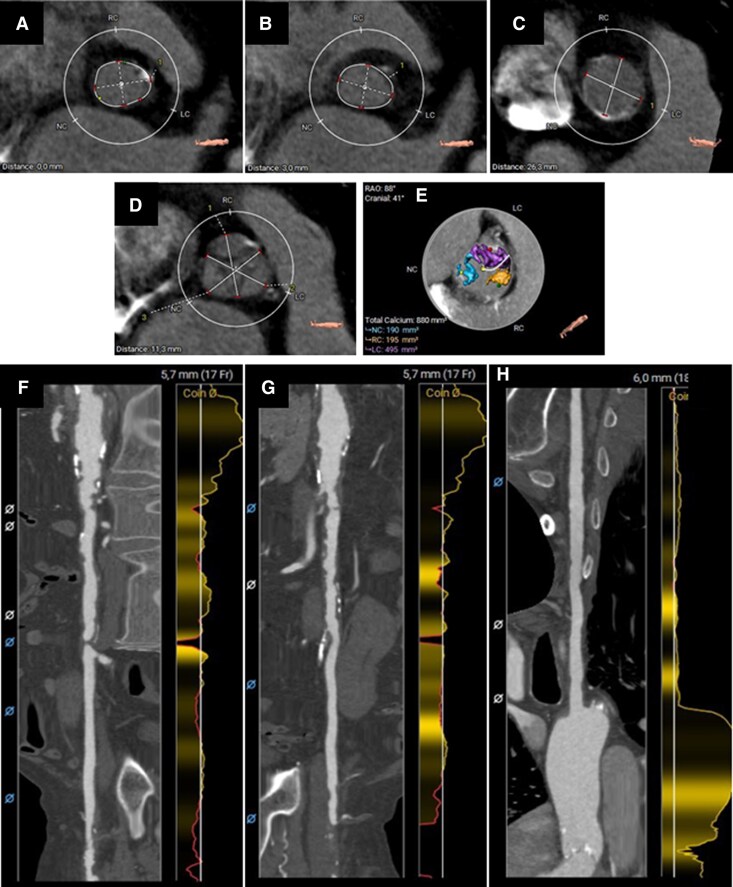
Computed tomography analysis. Panels are arranged from left to right and from top to bottom: *(A)* Aortic annulus sizing: area 376 mm^2^, perimeter 70 mm; *(B)* Left ventricular outflow tract sizing: area 365 mm^2^, perimeter 69 mm; *(C)* Sinotubular junction with mean diameter of 26.1 mm; *(D)* Aortic sinus measurements: right sinus 27.4 mm, left sinus 28.7 mm, non-coronary sinus 28.5 mm; *(E)* calcium distribution on aortic leaflets and annulus, *(F)* Right ilio-femoral axis with right common iliac artery occlusion; *(G)* Left iliac-femoral axis with occlusion of left common femoral artery just above the bifurcation; *(H)* Left subclavian and axillary axis.

## Procedural strategy

The procedural strategy was guided by three key constraints: the need for haemodynamic support in the setting of severe left ventricular dysfunction, avoidance of transvalvular device interaction during TAVI, and minimization of manipulation within the thrombotic aorto-iliac segment.

A newly developed sheathless iVAC-2L system was selected, representing, to our knowledge, its first clinical use. Compared with introducer-based systems and off-label sheathless approaches,^6^ it reduces the access profile to 16F and allows direct introducer-free insertion. The catheter is advanced over a stiff guidewire and positioned above the aortic leaflets, providing pulsatile support without crossing the valve and limiting interference during TAVI. Given the embolic risk associated with extensive aorto-iliac thrombosis, the MCS device was implanted via left subclavian access. A balloon-expandable Sapien 3 Ultra valve was selected to facilitate delivery beyond diseased segments and to accommodate the narrow sinotubular junction, while preserving the possibility of future coronary re-access.

Due to severe chronic kidney disease, continuous veno-venous haemofiltration (CVVH) was planned immediately after the procedure.

### Procedural execution

- Femoral and venous access preparation and iliac recanalization

Bilateral common femoral arterial access was obtained under ultrasound guidance, with pre-closure using two suture-mediated devices in the right femoral artery. The RCI occlusion was crossed with a 0.014″ guidewire and treated with percutaneous transluminal angioplasty (PTA) using a 5.5 mm balloon to restore patency. A 6F venous sheath was placed via the right femoral vein, and a temporary pacing lead was positioned in the right ventricle for rapid pacing during valve deployment.

- Subclavian protection and access

A JR4 catheter was positioned in the left subclavian artery, and a 0.035″ guidewire was advanced into the left brachial artery as a safety wire. The subclavian artery was punctured under fluoroscopic and ultrasound guidance, and two suture-mediated closure devices were pre-deployed. After placement of a stiff guidewire and vessel dilation with a 14F dilator, the 16F sheathless iVAC-2L catheter was advanced into the ascending aorta, positioned approximately 2 cm above the native leaflets, and pulsatile support was initiated.

- Protected left main–left anterior descending PCI

With the iVAC catheter in place, PCI of the LM–LAD was performed using a 7F EBU 3.5 guiding catheter and two drug-eluting stents (DES) with a provisional strategy. The patient remained haemodynamically stable throughout the PCI, including multiple balloon inflations for pre-dilatation, stent deployment, and post-dilatation.

- Transfemoral TAVI

After left coronary revascularization, the right external and common iliac arteries were further dilated with a 7 mm balloon. Following exchange for a stiff 0.035″ guidewire, a 14F, 36 cm eSheath + was advanced beyond the infrarenal thrombotic segments. The native valve was crossed and a Safari Small stiff wire was positioned in the left ventricular apex. A 23 mm Sapien 3 Ultra valve was advanced. Immediately before deployment, the iVAC-2L catheter was withdrawn approximately 5 cm to prevent balloon interaction, while mechanical support was maintained. Valve implantation was performed under rapid pacing (200 bpm). Final assessment showed a mean gradient of 8 mmHg with trivial paravalvular leak. The iVAC-2L was then repositioned above the prosthetic leaflets.

- Right coronary artery PCI

After TAVI, PCI of the RCA was performed using a JR4 guiding catheter through the existing eSheath+, with implantation of a DES from the ostium to the mid RCA. This sequence was chosen to avoid potential interaction between an ostial stent and the TAVI balloon.

- Haemostasis and access closure

After MCS weaning, protamine was administered and haemostasis of the subclavian access was achieved using the pre-deployed suture-based devices, with no vascular complications on angiography. Angiographic evaluation of the right iliac–femoral axis showed a satisfactory result after PTA, and haemostasis of the right femoral access was achieved with the pre-implanted closure sutures. Final angiography confirmed a good result of both PTA and access closure. Left femoral arterial and venous accesses were managed with manual compression.

All procedural steps are displayed in *Figures 2–3* and in Supplementary material online, *Videos S1*–S15.

**Figure 2:**
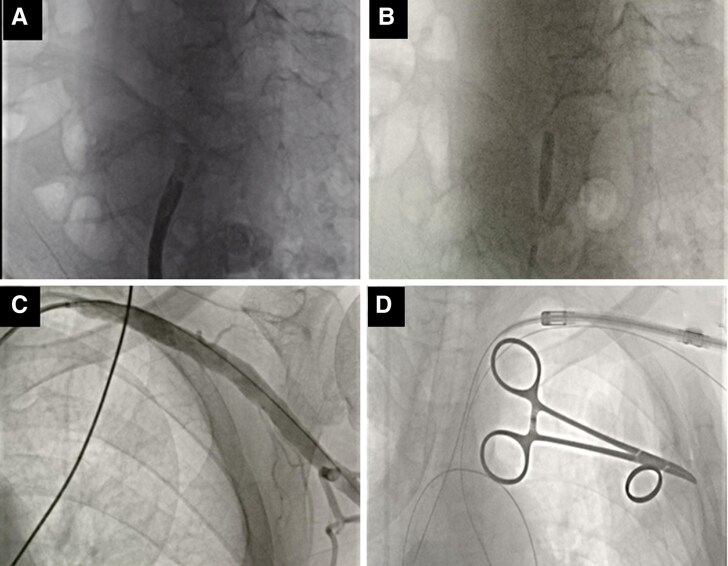
Procedural set-up with percutaneous transluminal angioplasty of right iliac occlusion and subclavian access for pulsatile sheathless support above the aortic leaflets. Panels are arranged from left to right and from top to bottom: *(A)* angiographic evidence of right common iliac artery occlusion; *(B)* percutaneous transluminal angioplasty of right common iliac artery; *(C)* basal angiography of left subclavian artery; *(D)* insertion of the dedicated sheathless iVAC-2L catheter.

**Figure 3:**
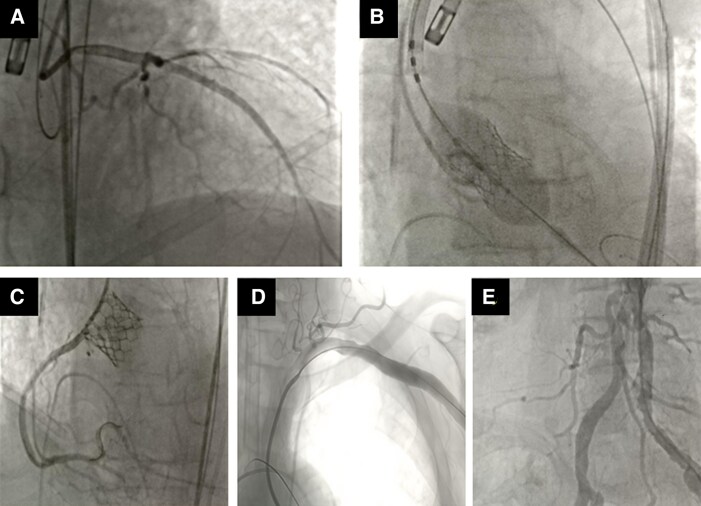
Pulsatile sheathless support above the aortic leaflets via subclavian access during complex percutaneous coronary intervention and transcatheter aortic valve implantation. Panels are arranged from left to right and from top to bottom: *(A)* result of left coronary artery percutaneous coronary intervention (provisional); *(B)* Sapien 3 Ultra 23 mm during rapid pacing; *(C)* result of right coronary artery percutaneous coronary intervention; *(D)* subclavian angiography after closure with two pre-implanted suture-based device, *(E)* right iliac-femoral axis after percutaneous angioplasty and closure with two pre-implanted suture-based device.

### Intra-procedural haemodynamics and post-procedural course

The patient remained haemodynamically stable throughout the procedure (mean arterial pressure 60–70 mmHg), without hypotension, escalation of inotropic support, or evidence of hypoperfusion. He was transferred intubated to the intensive care unit and extubated on postoperative day 1. No major bleeding, vascular complications, stroke, or limb ischaemia occurred. Doppler ultrasound was not performed because final angiography of the subclavian and ilio-femoral access sites showed preserved flow and no access-related complication. On serial clinical assessment, the access sites remained uncomplicated, distal pulses were preserved, and there were no signs of limb ischaemia or claudication after mobilization. Due to the administration of 250 mL of contrast, the patient's renal function was managed with CVVH, which was successfully discontinued after 8 days, with stable creatinine levels and no need for dialysis. At pre-discharge echocardiography, mean aortic gradient was 9 mmHg, paravalvular leak was trivial, and LVEF improved to 20%. The hospitalization timeline is reported in Supplementary material online, *Table S1*.

## Discussion

This case highlights the importance of tailoring access strategy and device selection to individual anatomical constraints. The sheathless iVAC-2L enabled 16F pulsatile support without transvalvular instrumentation, facilitating integration with balloon-expandable TAVI within a coordinated strategy balancing haemodynamic support and vascular protection. Despite the absence of complications, procedural risks should be considered. Extensive aorto-iliac thrombosis increases the risk of embolization and stroke, while subclavian access carries potential vascular complications. In addition, interaction between support devices and transcatheter valves remains a concern, particularly during deployment, underscoring the importance of careful planning and minimized vascular manipulation.

The same risk balance led the Heart Team to favour a single-session strategy, as staged procedures would have increased cumulative renal, vascular, and embolic risk in an already frail patient deemed at prohibitive surgical risk. The sequence of coronary treatment was also deliberate. LM–LAD PCI was performed before TAVI to protect the largest myocardial territory during valve implantation; because the left main ostium was not stented, there was no risk of interaction between the LM–LAD stent and the valve balloon. Conversely, RCA PCI was performed after TAVI to avoid potential deformation of an ostial RCA stent during balloon-expandable valve deployment.

Alternative MCS devices may be considered. Impella requires transvalvular positioning and may interfere with TAVI; VA-ECMO increases afterload and may enhance embolic risk in thrombotic aorto-iliac disease; similarly, to TandemHeart requires also a transseptal access. In contrast, iVAC-2L provides pulsatile supra-aortic support without crossing the valve, and its sheathless design reduces access size, representing a potential advantage in this setting.

## Conclusion

To the best of our knowledge, this is the first clinical report of the newly developed dedicated sheathless iVAC-2L catheter. While MCS has been used in high-risk PCI and TAVI, most devices require transvalvular positioning, potentially interfering with valve deployment. In this case, supra-aortic pulsatile support combined with a reduced access profile enabled a fully percutaneous, integrated PCI–TAVI strategy in the presence of complex coronary, valvular, and vascular disease. The sheathless design may represent an advantage in patients with severe peripheral arterial disease, where large-bore access is often a limiting factor.

## Supplementary Material

ytag502_Supplementary_Data

## Data Availability

The data underlying this article are available from the corresponding author upon reasonable request, subject to patient confidentiality.

## Lead author biography

Gioel Gabrio Secco, MD, PhD is an interventional cardiologist with expertise in complex coronary and structural heart interventions. His clinical focus includes high-risk percutaneous coronary intervention, mechanical circulatory support, and transcatheter aortic valve implantation. He is actively involved in clinical research and training, with particular interest in integrating haemodynamic support into complex procedures and optimizing strategies for anatomically challenging cases.

## Notes

**Consent**: Written informed consent was obtained from the patient for publication of this case report and accompanying material.
