# Supplementary material for: New sheathless supra-aortic pulsatile mechanical support via subclavian access for combined protected percutaneous coronary intervention and transcatheter aortic valve implantation in hostile vascular disease: a case report
Source: Eur Heart J Case Rep. 2026 Jul 10;10(7):ytag502. doi: 10.1093/ehjcr/ytag502 (PMC13398990; doi:10.1093/ehjcr/ytag502)
Supplement: ytag502_Supplementary_Data [file ytag502_supplementary_data.zip › Supplementary table 1.docx]

**Supplementary table 1: Timeline of patient’s hospitalization**

| **Timepoints** |  |
| --- | --- |
| Day 0 | Hospitalization in the ICCU for ADHF with evidence of severely depressed LVEF and severe aortic stenosis |
| Days 0 to 3 | Medical management with intravenous diuretics and dobutamine |
| Days 3 to 10 | Coronary angiography, computed tomography and Heart Team discussion |
| Day 10 | With trans-subclavian sheathless iVAC2-L support after PTA of right common iliac artery PCI of LM to LAD; TAVI with BEV and PCI of RCA |
| Day 10 to 18 | Medical management in ICCU and CVVH without inotropic support |
| Day 18 to 28 | Medical management in the Cardiology ward with physical therapy and discharge |

ICCU: intensive cardiac care unit; ADHF: acute decompensated heart failure; LVEF: left ventricular ejection fraction; PTA: percutaneous transluminal angioplasty; PCI percutaneous coronary intervention; LM: left main coronary artery; LAD: left anterior descending artery; TAVI: transcatheter aortic valve implantation; BEV: balloon expandable valve; RCA: right coronary artery; CVVH continuous veno-venous hemofiltration
